# Harvesting Costal Cartilage for Secondary Rhinoplasty: Techniques, Considerations, and Outcomes

**DOI:** 10.7759/cureus.69614

**Published:** 2024-09-17

**Authors:** Mohammed A Bagunaid, Mohammed A Borah, Abdullah M Abualjoud, Farss S Hariri, Hammad Nasir, Ka'ab M Ibrahim, Ali O Bajunaid, Eatedal M Al-Shareef

**Affiliations:** 1 Medicine, Batterjee Medical College, Jeddah, SAU; 2 Dermatology, JC Clinics, Jeddah, SAU

**Keywords:** cartilage grafting, patient satisfaction, rib cartilage, secondary rhinoplasty, surgical techniques

## Abstract

Secondary rhinoplasty is a surgical procedure aimed at reshaping the nose after the unsatisfactory results of primary rhinoplasty, which usually requires a reliable source of cartilage for grafting. Septal cartilage is usually the first option for rhinoplasty, but in cases where it is insufficient, rib cartilage might be used, which is the focus of the current study. The article details the surgical techniques for rib cartilage harvesting, highlighting the preference for the seventh rib done under general anaesthesia. The surgery requires a precise incision, harvesting of the cartilage, and careful closure while the cartilage is stored in a saline solution until use. It also introduces innovative approaches to minimize complications and improve patient satisfaction, including trans-umbilical endoscopic harvesting, the use of the 10th rib to reduce morbidity, and employing fresh frozen homologous rib cartilage as a grafting alternative. Based on various studies, most cases following rhinoplasty with rib cartilage showed a high satisfaction rate despite the complexity of the procedure. Patient consideration includes good communication with realistic expectations through imaging techniques. We also discuss complications, which can heavily impact the patient's quality of life. We hope that by providing this information, our paper will provide surgeons and researchers with the latest information on this topic.

## Introduction and background

Rhinoplasty is a surgical procedure for altering the nose. It can be either reconstructive to restore form and function of the nose or cosmetic to change its appearance making it more aesthetically pleasing. Rhinoplasty can further be divided into two types, primary and secondary. Primary rhinoplasty is the initial reconstructive or aesthetic procedure, whereas secondary rhinoplasty is the reoperation of a previously operated nose. The main reasons for a secondary rhinoplasty are complications of the initial surgery, an undesirable or unsatisfactory result from the primary surgery and the need for additional improvement. Therefore, another common name for secondary rhinoplasty is revision rhinoplasty. Revision rhinoplasties are not uncommon, as 5% to 20% of primary rhinoplasties end up requiring revision [1,2].

When compared to primary rhinoplasties, secondary rhinoplasties are significantly more complex and challenging. This is due to multiple factors such as the presence of scar tissue, the disruption of anatomical structures and an insufficient and therefore destabilized osseocartilaginous framework, likely due to extensive reduction and resection in the initial surgery. Furthermore, another source of difficulty is that the patients’ expectations are higher due to the fact they are disappointed by the initial surgery. Additionally, the aesthetic outcome desired by the patient may lead to breathing difficulties caused by insufficient central supports and narrowing of the airways. Therefore, secondary rhinoplasty requires a broader repertoire of surgical management skills to convert an unhappy patient to a satisfied one [3,4].

The outcome of the procedure is affected by multiple variables such as the length of the nasal bone as well as the strength and resiliency of the cartilage. As a result, secondary rhinoplasty frequently involves the use of extra-nasal donor material to create a stable cartilaginous framework and to achieve the desired aesthetic result. Ideally, the graft material should be sufficiently strong, resilient and of adequate quantity. The current gold standard for revision rhinoplasty is the use of autologous grafts. Autologous grafts can be sourced from five potential areas: septal cartilage, auricular cartilage, and rib cartilage, as well as iliac and calvarial bone [3-5].

This article will focus on the use of costal cartilage as the grafting material. Autologous costal cartilage has lower risk of graft harvest morbidity, infection, warping and extrusion. Moreover, costal cartilage grafts offer significant versatility in terms of shape, length, and width, enabling nasal reconstruction to meet nearly any functional or aesthetic need. The seventh rib is the most ideal source for the graft as its cartilage is straight, of ample quality and quantity, located far from the pleural cavity and can be harvested with minimal donor site morbidity [3-5].

## Review

Patient considerations/selection

Dealing with the secondary rhinoplasty patient requires more skill and patience than a primary case. Patients need greater assurance than they did before their first surgery, and they are frequently dissatisfied and doubtful. The operation is often more complicated but not necessarily so. It is important to acknowledge the patient’s complaints for each patient's preoperative planning. Cameras are affordable and generally accessible. By showing the patient what you believe needs to be done and getting their input on what they believe should be done as well, you can generally reach some kind of consensus. The patient must understand what is and is not achievable. The surgeon discovers any potential issues during the imaging process. The process of modifying a result on the monitor can occasionally provide valuable insight [6].

In order to better comprehend the patient's aesthetic problems and facilitate the completion of the questionnaire, the nose is divided into upper, middle, and lower thirds. The nasal issues in the upper and middle areas such as high or low, broad or narrow, crooked, irregularity of nasal bridge, or other changes are classified in the interview. Nose bulbous tip, narrow/pinched tip, and upturned/raised tip are a few issues with the lower third. Asymmetrical, lacking appropriate tip definition, and collapse during inspiration and other changes are mentioned by those interviewed [1].

Radiology

The costal cartilage width at the level of the medioclavicular line on both right and left side was measured using frontal CT (computed tomography) images. The thickness of each individual rib and the soft tissue envelope anterior to each individual rib at the level of the medioclavicular line were measured using axial CT images. The length, calcification, and soft tissue envelope of the costal cartilage on the left and right sides were compared. Some authors advise against harvesting costal cartilage from the left side due to the possibility of pleura or pericardium tears [7].

According to some authors, the right side is preferred since chest pain on the left side can be mistaken for heart-related pain throughout the healing phase. Nonetheless, the left side of the patients under study had the highest concentration of costal cartilage from the sixth, seventh, and eighth ribs. Further investigation showed that the only patients with more costal cartilage on the right side were those between the ages of 31 and 50 while there was not a noticeable distinction between the older and younger individuals. For individuals who have had surgery, radiation therapy, or trauma resulting in the loss of costal cartilage on the right side, rib cartilage harvesting from the left side is still an option. Gender and the thickness of the soft tissue envelope don't seem to be important considerations for the surgical approach [7].

Conventional surgical techniques for rib cartilage harvesting

Harvesting rib cartilage is done while the patient is lying in a supine position and under general anaesthesia. The graft of rib cartilage could be harvested either from the patient’s right side or left side, but it is preferred to be harvested from the left side to support a two-team approach. Rib marking begins by palpating the angle of Louis, which identifies the location of the second rib. Subsequent ribs are then palpated and numbered until the fifth, sixth, and seventh ribs are reached. Those ribs are usually considered due to their depth, length, and position. The seventh is most often selected for the graft [8,9].

**Figure 1:**
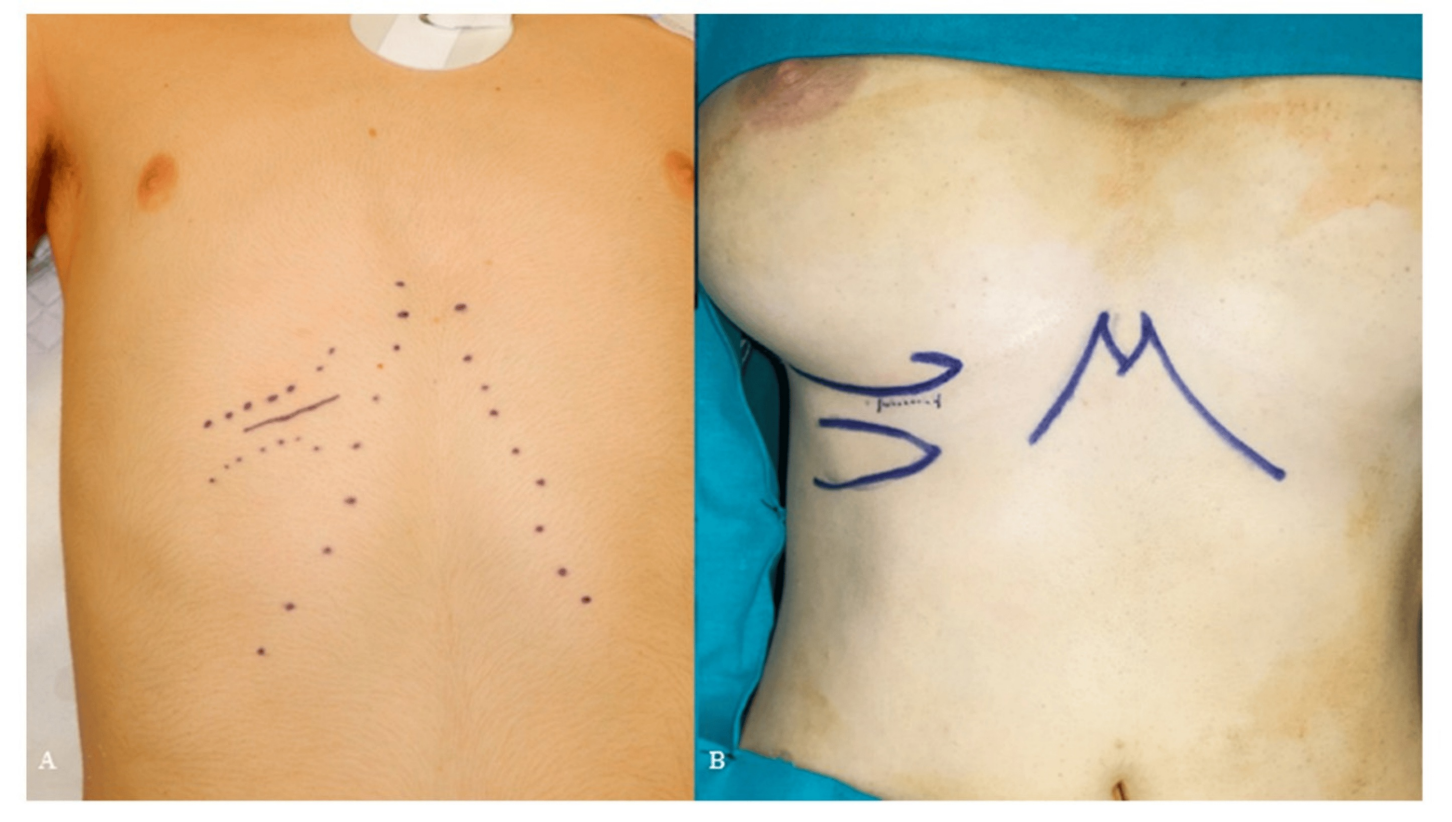
(A) surgical marking incision in a male patient, (B) surgical marking incision in a female patient. Reproduced under open access Creative Common CC BY license from [10]

In male patients, the incision is marked directly over the chosen rib to facilitate the dissection (Figure 1A). In female patients, the incision is made approximately 5 mm above the inframammary fold and is about 5 cm long (Figure 1B). It is important to avoid extending the incision beyond the medial boundary of the inframammary fold to ensure minimal visibility post-operatively [5,8].

**Figure 2:**
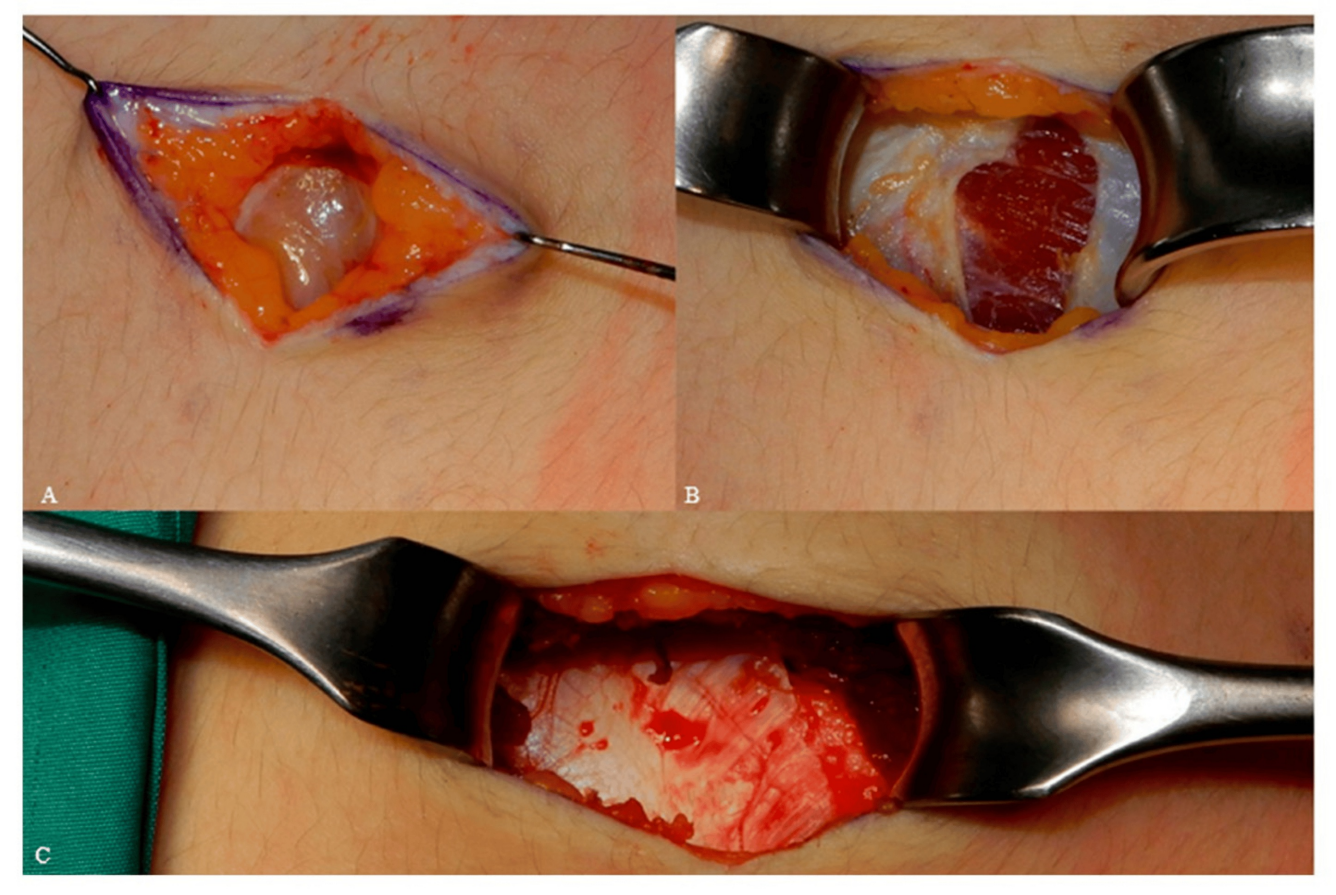
(A) incising skin and fat, (B) incising fascia, (C) muscle incision. Reproduced under open access Creative Common CC BY license from [10]

After deciding the placement of the incision, the harvesting process begins with a skin incision made using a number 15 blade. Subcutaneous tissue and fascia are transacted using electrocautery by the use of retractors, until adequate surgical view is achieved (Figure 2). After reaching the muscle fascia, it's crucial to palpate the underlying ribs to ensure the dissection follows the longitudinal axis of the selected rib accurately. A lengthwise incision is made between the rectus abdominus muscle and external oblique muscle. The muscles are then dissected in a direction parallel to their fibres [5].

Once the chosen rib is exposed, a longitudinal incision is done using electrocautery through the perichondrium along the length of the rib’s central axis. On the medial and lateral aspect of the cartilaginous rib, perpendicular incisions are performed to separate and lift the perichondrium easily. The dissection is then extended medially until the junction between the rib cartilage and sternum is palpable. The dissection is limited laterally by the costochondral junction. The bony rib is generally reddish-grey hue, while the cartilaginous part is off-white [5,10].

The perichondrium is elevated superiorly and inferiorly from the cartilaginous rib using a periosteal elevator. Next, the dissection is carried circumferentially along the length of the cartilaginous portion of the rib until the posterior aspect of the rib is revealed. If the perichondrium tightens during elevation, making perpendicular incisions on its anterior surface can help alleviate this tension. Next, the posterior adherence between the perichondrium and cartilage is released using a perichondrial elevator, and completing the posterior dissection by sliding the rib stripper back and forth along the rib until weakening is complete [5,10,11].

**Figure 3:**
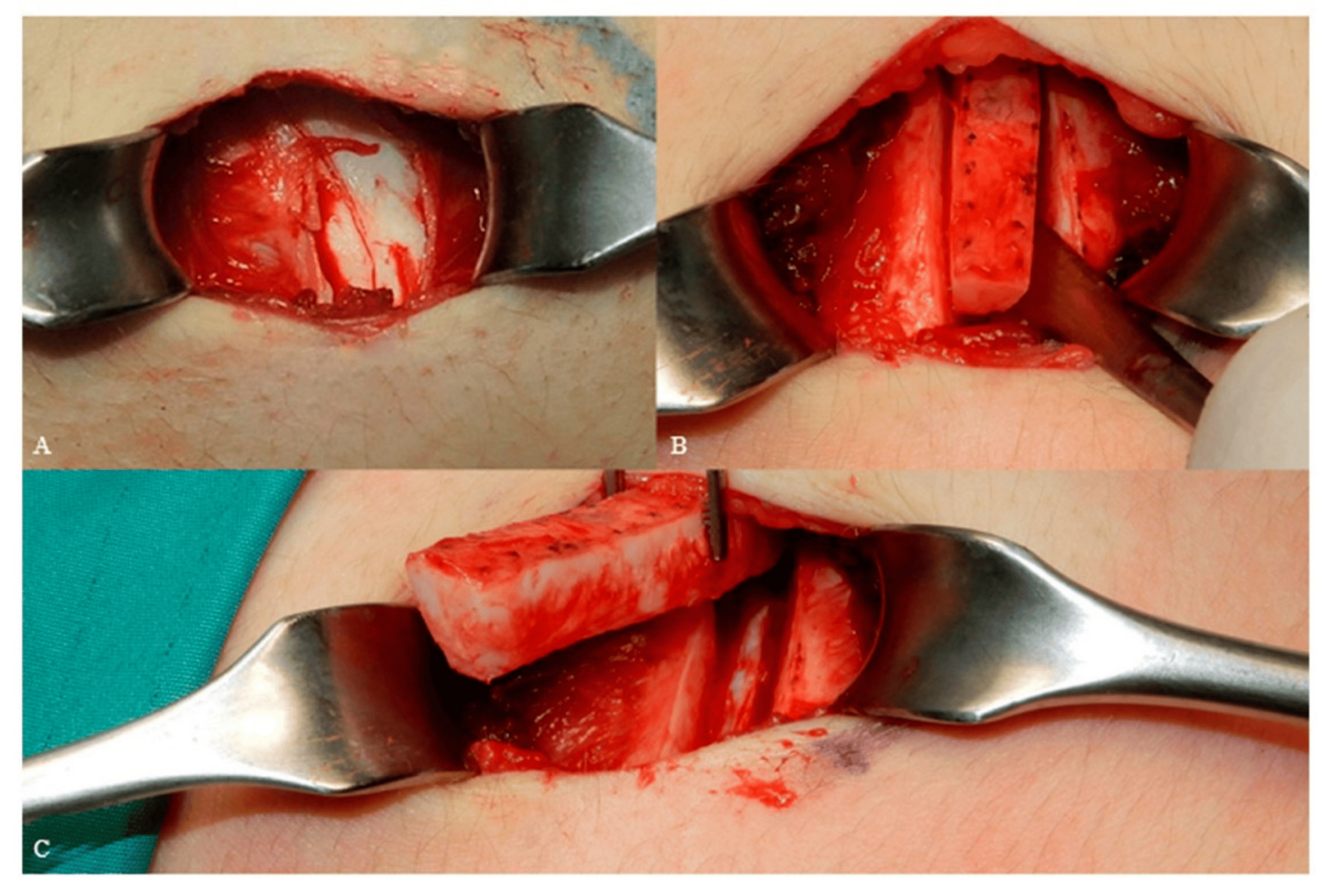
(A) Exposing the cartilage, (B) incising the cartilage, (C) harvesting the rib cartilage. Reproduced under open access Creative Common CC BY license from [10]

Finally, detaching the cartilaginous rib from its medial connection near the sternum and its lateral attachment at the bony rib, making perpendicular incisions to the long axis of the rib by the use of number 15 blade. The incision of the cartilage can be finished by using the end of a freer elevator, gently moving it from side to side (Figure 3) [10]. 

After releasing the cartilage segment from both sides, medially and laterally, the graft is extracted easily and placed into a sterile saline solution with gentamicin, 50 mg/500 cc till fabrication [5,10,11].

**Figure 4:**
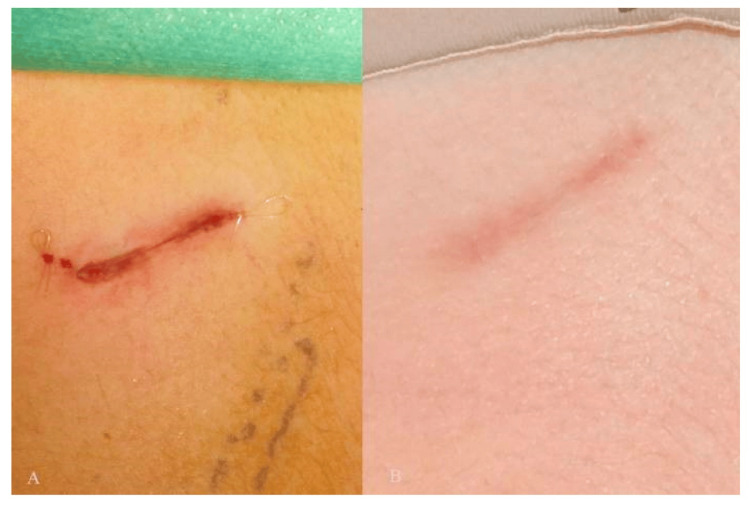
Post-surgical suturing (A), six months after the procedure (B). Reproduced under open access Creative Common CC BY license from [10]

After the control of bleeding and achievement of haemostasis, the scientist checks by a leak test to verify the absence of pneumothorax. It is done by filling the wound with saline and asking the anaesthesiologist to apply positive pressure into the lungs. Excluding pneumothorax is confirmed if no air leak is detected [11,12]. The wound is closed in layers. It is important to close the perichondrium tightly, as this rigid layer helps splint the wound and reduces postoperative pain. Wound closure is carried out by approximating the muscle and facial layers with a 2-0 vicryl suture. Skin closure is completed by the use of deep dermal and subicular four-zero monocryl sutures (Figure 4). Post-surgical pain management is performed by injecting 10 cc of 0.75% ropivacaine at the donor site [5,10].

New innovative techniques

Although the costal cartilage is a popular choice for autologous grafting in secondary rhinoplasty, its use can be complicated with donor site morbidities such as pneumothorax, deformities of the chest wall, post-operative pain and infections. As such, new methods and techniques are constantly being researched to circumvent these complications and to obtain a higher level of patient satisfaction. Three such new techniques, which include trans umbilical endoscopic costal cartilage harvesting, using fresh frozen homologous rib cartilage and the use of 10th rib cartilage, will be briefly discussed [12].

Harvesting the costal cartilage conventionally involves an incision on the anterior-medial chest wall over the donor site. This leaves behind a scar which can be significantly concerning for the patient. One new approach, proposed by authors Ching and Hsiao (2014) for harvesting the costal cartilage via a trans-umbilical endoscopic approach hopes to avoid such scarring. In this technique the endoscope is introduced through an umbilical incision and moved through a tunnel, which is created by superficial dissection and electrocautery, towards the donor site. There were no associated complications after the surgeries and the authors concluded that this new technique provided an effective alternative for costal cartilage harvesting, only leaving behind an unremarkable scar hidden by the umbilicus [12].

While the seventh rib is a common choice for autologous cartilage harvesting, it is still associated with some disadvantages. When harvesting the cartilage of the seventh rib a large incision is required as the rectus abdominus muscle (RAM) drapes over the cartilage. This results in increased donor-site morbidity and post-operative pain [13].

Authors Kim et al. (2015) proposed the use of the 10th rib cartilage to minimize these disadvantages. The 10th costal cartilage was chosen as its position above the abdominal cavity of the inferior thorax and above the diaphragm and transverse abdominis muscle lowers the risk of pneumothorax. Furthermore, its connection to the costal arch means that it can easily be located and can be harvested with the ninth rib should the need arise. Additionally, since the 10th costal cartilage is lateral to the RAM, a large incision is not required to harvest the cartilage which results in decreased donor-site morbidity and post-operative pain. Thus, the authors concluded that the 10th costal cartilage was a safe and effective alternative for rhinoplasty [13].

Another novel approach involves the use of fresh frozen homologous rib cartilage (FFRG) as a substitute for autologous rib cartilage. A review by Salzano et al. (2024) concluded that FFRG use resulted in decreased donor site morbidities such as pneumothorax and shorter operating times [14].

Complications

All postoperative visits showed a decrease in donor site pain scores over time, but there were no statistically significant variations in general pain scores. At one week, the values for rib cartilage grafts were significantly higher than those for ear cartilage grafts in terms of nasal pain. This noteworthy distinction between the two groups persisted one month after surgery. But three months following the procedure, the amount of nose pain had not decreased much [15].

Regarding scar itching, there were no appreciable differences between patients who had rib cartilage grafts and those who had ear cartilage grafts. Over time, fewer patients reported experiencing scar itching. Over time, fewer patients reported a change in scar colour, thickness, or stiffness for either group; however, at different stages following surgery, there were several cases where there was a notable difference between the two groups [15].

The main complications that arise from rib harvesting at the donor site include pneumothorax, infection, seroma, scarring, and postoperative pain. The most common donor-site problem, according to the most recent research on the subject, is scarring; temporary pain at the donor site is not considered an issue [16].

Postoperative pain at the donor site is common with chest wall pain being the most common. Following the first week, the pain at rest goes away, but patients still have severe discomfort several weeks following the procedure when it comes to movement. In the postoperative period, the majority of patients stop doing any activity and act as though they had suffered a serious localised rib injury. Therefore, quality of life is severely reduced, especially in the early postoperative period with chest wall pain being prevalent [16].

Following the first week, the pain at rest goes away, but patients still have severe discomfort for several weeks following the procedure when it comes to movement. Most patients refrain from activities and act as though they have had a serious localised rib injury during the recovery phase. Consequently, there is a significant reduction in quality of life, particularly in the early postoperative phase [16].

Outcomes and patient satisfaction

In most cases, there were no complications following rhinoplasty, and the results were great. Moon et al. in 2012 used autologous costal cartilage for dorsal grafts in 80 patients form 108 research individuals. The results of the study showed that the outcomes of rhinoplasty were satisfactory in 37 instances, bad in four cases, and excellent in 43 cases. According to the patient-reported subjective satisfaction surveys, 73 patients reported feeling content, 16 reported feeling better than they did before surgery, and 19 reported being dissatisfied. The patient's complaints were attributed to a number of factors, including a columellar scar (n = 6), low radix and dorsum (n = 5), nostril asymmetry (n = 3), remnant nasal deviation (n = 3), progressive nasal deviation (n = 1), and high radix (n = 1). Of the 19 patients who were not satisfied, nine underwent revision rhinoplasty and two underwent scar revision, and those 11 patients had revision rhinoplasties to treat uncorrected congenital nostril stenosis (n = 1), graft resorption (n = 5), graft fracture following nasal trauma (n = 2), warping (n = 1), a high nasal dorsum (n = 1), and remnant nasal deviation (n = 1), even though other patients than the 19 unhappy patients had requested minor adjustments. Thirteen patients experienced complications at the donation site. A seroma developed in nine of the patients' chest wounds a few days following surgery. A pneumothorax occurred in one case. The incision on the chest healed into keloid scars in two individuals. One patient experienced persistent pain at the site of the chest incision. Nineteen individuals had recipient site problems. Nine of them had infections, five had graft resorption, two had visible graft contour, two had graft fracture due to nasal trauma, and one had warping [16].

In another study, Cakmak and Ergin (2002) reviewed 20 rhinoplasty patients who had their nose reconstructions made using costal cartilage. Follow-up periods ranged from eight to 32 months. One patient experienced an early wound infection in the columellar region, three patients experienced mild tissue warping, and there were no instances of graft resorption. The authors concluded that autogenous costal cartilage is an outstanding material for support and volume filling in rhinoplasty procedures [17].

In another study done by Loghmani et al. in 2019, the authors noted that following oblique split rib graft surgery, 25 patients underwent primary and secondary septorhinoplasty after three- to 36-month follow-up period. All the patients had successful surgical outcomes and no signs of severe resorption, infection, warping, or displacement. The patients were also entirely satisfied. When the donor-site morbidity was objectively evaluated, each case had scars of high quality and no problems. Following surgery, none of the patients experienced any problems, and throughout the follow-up period, none of the patients reported nasal distortion [18]. 

## Conclusions

Despite its complexity and high patient expectations, secondary rhinoplasty has a high success rate. New techniques of harvesting the costal cartilage are constantly being researched to circumvent certain complications associated with the conventional technique and to give better patient satisfaction. Nasal pain is significantly higher with rib cartilage grafts than with ear cartilage grafts. Complications of the donor site nonetheless include pneumothorax, infection, seroma, scaring and post-operative pain. Patients frequently request minor changes post-operatively despite expressing their satisfaction.
